# Evaluation of MicroRNA 145 and MicroRNA 155 as Markers of Cardiovascular Risk in Chronic Kidney Disease

**DOI:** 10.7759/cureus.66494

**Published:** 2024-08-09

**Authors:** Amit Kumar, G Priyadarshini, Sreejith Parameswaran, Ananthakrishnan Ramesh, Medha Rajappa

**Affiliations:** 1 Department of Biochemistry, Jawaharlal Institute of Postgraduate Medical Education and Research, Puducherry, IND; 2 Department of Nephrology, Jawaharlal Institute of Postgraduate Medical Education and Research, Puducherry, IND; 3 Department of Radiodiagnosis, Jawaharlal Institute of Postgraduate Medical Education and Research, Puducherry, IND

**Keywords:** flow mediated dilatation, endothelial dysfunction, micro-rna 155, micro-rna 145, chronic kidney disease

## Abstract

Background

Chronic kidney disease (CKD) leads to a progressive decline in renal function, primarily due to deteriorating kidney structures. Vascular calcification is a key effect of CKD. MicroRNAs (miRNAs) play a significant role in the onset and progression of both cardiovascular illness and CKD.

Aim

The aim of this study was to compare biomarkers of endothelial dysfunction, 25-hydroxyvitamin D (25(OH)D), intact parathyroid hormone (iPTH), miRNA 155, and miRNA 145, in patients with CKD versus controls.

Methods

We recruited 60 patients with CKD and 60 controls. All participants underwent brachial artery flow-mediated dilatation (FMD). Asymmetric dimethylarginine (ADMA) levels were measured using ELISA. Levels of miRNA 145 and miRNA 155 were quantified using real-time polymerase chain reaction (PCR).

Results

Serum levels of miRNA 145, miRNA 155, 25(OH)D, and FMD were significantly lower in CKD patients compared to controls. Conversely, serum ADMA and iPTH levels were significantly higher in CKD patients. There was a significant negative association between miRNA 145, miRNA 155, FMD, and 25(OH)D with ADMA and iPTH. Additionally, miRNA 145, miRNA 155, FMD, and 25(OH)D showed a significant positive correlation with estimated glomerular filtration rate (eGFR) and with each other.

Conclusion

Lower levels of miRNA 145 and miRNA 155 and increased endothelial dysfunction correlate with CKD severity, suggesting an accelerated risk for cardiovascular disease (CVD).

## Introduction

Chronic kidney disease (CKD) is a condition characterized by an irreversible loss of renal function, accompanied by imbalances in calcium, phosphate, parathyroid hormone (PTH), and vitamin D levels [1]. These imbalances lead to disruptions in mineral and bone metabolism and predispose individuals to vascular calcification (VC) and cardiovascular disease (CVD) [2]. CVD is the leading cause of mortality and morbidity in end-stage renal disease (ESRD) patients [3]. One of the contributing factors to CVD is the disintegration of the vascular structure due to endothelial dysfunction.

Asymmetric dimethylarginine (ADMA) increases endothelial cell dysfunction, contributing to the progression of atherosclerosis [4] by inhibiting endothelial nitric oxide synthase (eNOS) and reducing the production of nitric oxide (NO) in endothelial cells. The gold standard for assessing endothelial dysfunction is flow-mediated dilatation (FMD) in the brachial artery, which measures the endothelium's capacity to respond by releasing NO [5]. Endothelial dysfunction impacts FMD by impairing the endothelium's ability to synthesize NO. Measuring endothelial dysfunction is a non-invasive early method of assessing CVD, as it progresses in direct proportion to the course of CKD [6].

MicroRNAs (miRNAs) are short, non-coding RNAs, limited to 25 nucleotides in length, that affect post-transcriptional gene expression [7]. MiRNAs contribute to the onset and progression of CKD and the elevated cardiovascular (CV) risk experienced by these individuals [8]. Recent research has linked miRNA 145 [9] and miRNA 155 [10] to coronary artery disease and vascular damage.

MiRNA 145 is derived from the chromosome 21 B-cell integration cluster (BIC) and regulates the development of vascular smooth muscle cells (VSMCs) [11]. It is the most prevalent miRNA in vascular walls [12], with its expression increasing as vascular damage progresses [11]. MiRNA 155 is transcribed from a 4.09 kb region on human chromosome 5 (5q32-3) [13]. Research has shown that miRNA 155 is associated with anomalies in the angiotensin pathway [14] and estimated glomerular filtration rate (eGFR) [15].

Our team has previously investigated the connection between endothelial dysfunction and the severity of CKD [16]. Given the crucial role miRNAs play in gene expression, we aimed to explore the relationship between miRNAs and the risk of CVD as biomarkers for both CKD and its complications, such as CVD. To achieve this goal, we assessed the levels of miRNA 145, miRNA 155, intact parathyroid hormone (iPTH), 25-hydroxyvitamin D (25(OH)D), and markers of endothelial dysfunction (ADMA and FMD) in patients with CKD.

## Materials and methods

Study design

This cross-sectional study was conducted from January 2020 to March 2021 at a tertiary care center in Puducherry. The study received approval from the Institute Ethics Committee (Project no: JIP/IEC/2019/391), and informed consent was obtained from all participants. The study was conducted following the 2017 ICMR National Biomedical Guidelines for Research in Human Participants.

Sample size calculation

To detect an effect size of 0.3 among pairs, a total of 60 samples (number of pairs) were required to achieve a power if 80% and a significance level of 5% (two-sided), based on prior literature in the field [17]. Therefore, the sample size was 120, comprising 60 patients with CKD and 60 healthy controls. 

Study population

The study included 60 age- and gender-matched healthy control volunteers and 60 pre-dialysis non-diabetic CKD patients (aged 18-70 years) from South India, recruited from the nephrology department of a tertiary care facility. Participants with current infections or inflammatory processes, pre-existing peripheral vascular disease or atherosclerotic vascular disease, cancer, diabetes mellitus, and pregnant women were excluded from the study. Diabetic individuals were excluded due to their higher likelihood of experiencing CVD complications from CKD.

Assessment of study parameters

For all participants, fasting blood glucose, lipid profile, creatinine, urea, calcium, phosphorous, 25(OH)D, and iPTH levels were obtained from the case records. The Chronic Kidney Disease Epidemiology (CKD-EPI) equation was used to estimate GFR and determine the severity of CKD (in mL/min/1.73 m^2^) [18]. Blood samples (5 mL) were collected in EDTA-coated tubes. Plasma was isolated and stored at -80°C. Total RNA, including miRNA, was extracted using a commercially available kit (miRNeasy serum/plasma isolation kit, Qiagen, Germany). The extracted miRNA was converted into cDNA using TaqMan advanced miRNA cDNA synthesis kit (Applied Biosystems, Waltham, Massachusetts). Quantification of miRNA 145 and miRNA 155 was performed by quantitative real-time PCR (Bio-Rad Laboratories Inc., Hercules, California) using TaqMan advanced miRNA assays (Applied Biosystems), with Cel-miRNA 39-3p as the control gene. Samples were quantified in duplicates, and miRNA expression levels were analyzed using the 2-ΔΔCT method. ADMA concentration was determined by enzyme-linked immunosorbent assay (ELISA) (Bioassay Technology, Inc., China).

Assessment of endothelial dysfunction by flow-mediated dilatation

A Prosound Alpha 6 color Doppler ultrasound scanner (Aloka, Japan) with a 9 MHz linear array transducer was used to perform FMD of the brachial artery. Participants were instructed to fast for six to eight hours prior to the procedure. The brachial artery was scanned with the individual in supine posture. At least a day before the procedure, participants were adviced not to smoke, drink alcohol, or consume any medications that might impair endothelial function. The probe was carefully positioned in a longitudinal section just above cubital fossa, over the brachial artery of the left upper limb. The echogenic intimal layers of the brachial artery were displayed, and the width of the brachial artery was measured at baseline between the near and far intimal layers. Three readings were taken, and the mean was used to determine the baseline diameter. A blood pressure cuff was tied around the upper forearm and inflated to a pressure 50 mmHg above the participant's systolic blood pressure, which was maintained for 5 minutes. After 5 minutes, the cuff was released. The width of the brachial artery was measured above the cubital fossa one minute after the cuff was released. The mean of the three readings was used to determine the post-FMD diameter. The formula used to measure FMD, also known as proportional FMD, is given as follows:

FMD = [(post-FMD diameter - baseline diameter)/baseline diameter] x 100

Statistical analysis

IBM SPSS Statistics for Windows, version 20 (Released 2011; IBM Corp., Armonk, Newyork) and GraphPad Prism (San Diego, California) for Windows were used for the statistical analysis. The Kolmogorov-Smirnov test was used to determine the normality of the data. Percentages and frequencies were used to describe categorical variables, and they were compared using the chi-square test. Mean with standard deviation or median with interquartile range was used to express continuous variables and compared by the independent Student's t-test or a Mann-Whitney U test, as appropriate. A comparison of variables between low and high CV risk was done among cases (based on FMD, a surrogate marker of CV risk) [19]. The association between ADMA, FMD, 25(OH)D, iPTH, and miRNA levels with disease severity was studied using Spearman rank correlation. Statistical analysis was done at a 5% level of significance, with p<0.05 considered statistically significant.

## Results

Sixty individuals with CKD and 60 controls who were age- and gender-matched were enrolled in the study. There were 42 males and 18 females in each group. Compared to the control group, the CKD group had a significant increase in systolic blood pressure and a significant drop in eGFR. The CKD group had a 36-month median disease period (Table 1).

**Table 1:** Comparison of baseline characteristics between cases and controls ^#^Mann-Whitney U test.

Parameter	Cases (n=60), median (IQR)	Controls (n=60), median (IQR)	U-value	p-value ^#^
Age (years)	48.00 (43.00-53.00)	44.00 (37.00-51.00)	1593.00	0.27
Gender (M:F)	42 (70%):18 (30%)	42 (70%):18 (30%)	--	--
Body mass index (kg/m^2^)	22.65 (21.95-23.33)	22.77 (21.79-23.37)	1740.00	0.75
Waist-hip ratio (W:H)	0.97 (0.94-0.97)	0.97 (0.94-0.97)	1751.50	0.79
Systolic blood pressure (mmHg)	130.00 (128.00-140.00)	122.00 (120.00-128.00)	758.00	<0.0001
Diastolic blood pressure (mmHg)	80.00 (78.00-90.00)	80.00 (80.00-86.00)	1793.50	0.97
Estimated glomerular filtration rate (mL/min/1.73m^2^)	17.6 (15.40-31.00)	86.05 (80.70-102.10)	0.000	<0.0001
Duration of chronic kidney disease (months)	36.00 (24.00-48.00)	--	--	--

While comparing baseline biochemical parameters, serum creatinine, urea, total cholesterol, very low-density lipoprotein, low-density lipoprotein, triglycerides, high-density lipoprotein, calcium, and phosphorus levels were observed to be significantly higher in the cases compared to controls (Table 2).

**Table 2:** Comparison of routine biochemical parameters between cases and controls *Independent Student’s t-test. ^#^Mann-Whitney U-test.

Parameter	Cases (n=60), mean±SD/median (IQR)	Controls (n=60), mean±SD/median (IQR)	t-value/U-value	p-value
Fasting blood sugar (mg/dL)	83.50 (74.50-89.00)	82.00 (76.25-88.75)	1775.50	0.89^#^
Creatinine (mg/dL)	3.70 (2.20-4.00)	0.90 (0.90-1.10)	0.000	<0.0001^#^
Urea (mg/dL)	92.00 (79.00-120.25)	21.00 (17.00-24.00)	0.000	<0.0001^#^
Total cholesterol (mg/dL)	197.00 (180.25-220.50)	151.00 (147.00-183.00)	690.00	<0.0001^#^
High-density lipoprotein (mg/dL)	48.40 ± 12.33	43.33 ±11.69	-2.31	0.02*
Low-density lipoprotein (mg/dL)	109.90 (100.60-133.80)	91.80 (76.45-108.85)	911.50	<0.0001^#^
Very low-density lipoprotein (mg/dL)	34.40 (27.20-47.40)	19.20 (14.80-23.60)	642.50	<0.0001^#^
Triglycerides (mg/dL)	172.00 (136.00-237.00)	96.00 (74.00-118.00)	642.50	<0.0001^#^
Calcium (mg/dL)	8.80 (8.50-9.20)	8.75 (8.20-9.00)	1419.00	0.04^#^
Phosphorus (mg/dL)	6.00 (4.20-6.77)	2.75 (2.50-3.10)	78.00	<0.0001^#^

**Figure 1:**
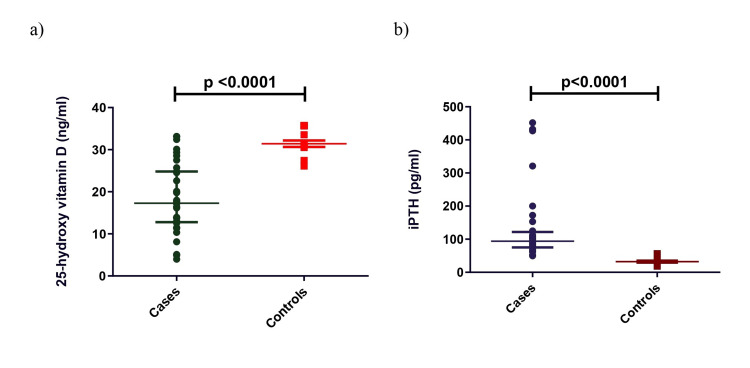
Comparison of 25-hydroxyvitamin D (a) and intact parathyroid hormone (b) in CKD cases and controls. Mann-Whitney U-test was used to analyze these data. iPTH: intact parathyroid hormone, CKD: chronic kidney disease.

**Figure 2:**
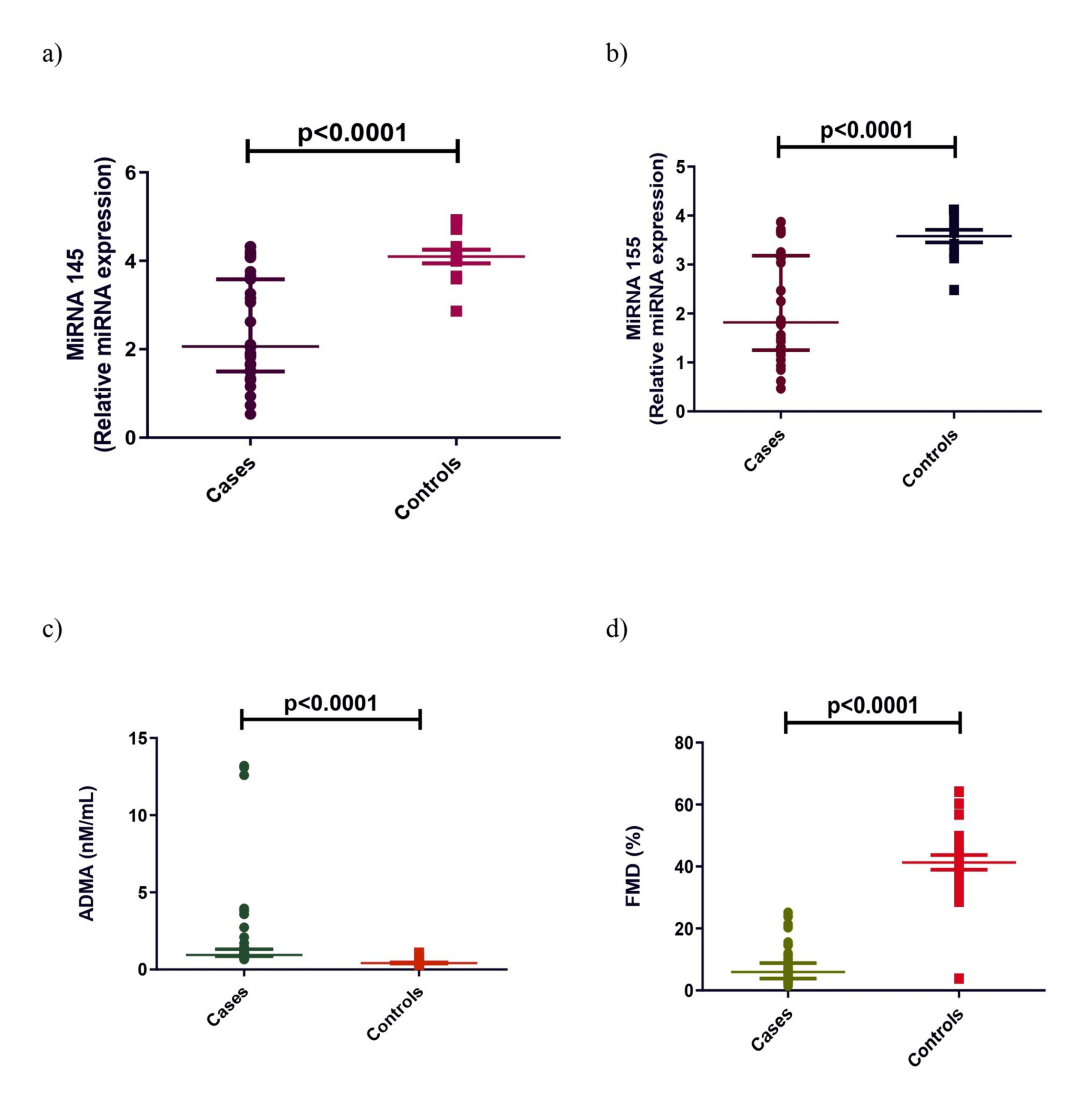
Comparison of miRNA 145 (a), miRNA 155 (b), ADMA (c), and FMD (d) in CKD cases and controls. Mann-Whitney U-test was used to analyze these data. ADMA: asymmetric dimethylarginine, FMD: flow-mediated dilatation, CKD: chronic kidney disease, miRNA: microRNA.

Plasma 25(OH)D, miRNA 145, miRNA 155, and FMD levels in CKD patients were significantly lower than in controls. In contrast, plasma iPTH and ADMA levels were significantly higher in cases compared to controls (Figures 1, 2).

The correlation between study parameters is shown in Table 3. It was observed that miRNA 145, miRNA 155, FMD, and 25(OH)D have a significant negative correlation with ADMA and iPTH. ADMA and iPTH have a significant positive correlation with each other. miRNA 145, miRNA 155, FMD, and 25(OH)D were found to have a significant positive correlation with each other and with eGFR. ADMA and iPTH had a significant negative relationship with eGFR. CKD duration did not exhibit any correlation with any of the study parameters.

**Table 3:** Correlation of study parameters* *Spearman rank correlation.

Parameter	Asymmetric dimethylarginine		miRNA 145		miRNA 155		Intact parathyroid hormone		25-hydroxyvitamin D		Flow-mediated dilatation		Estimated glomerular filtration rate		Duration of CKD	
	Rho value	p-value	Rho value	p-value	Rho value	p-value	Rho value	p-value	Rho value	p-value	Rho value	p-value	Rho value	p-value	Rho value	p-value
Asymmetric dimethylarginine	-	-	-0.664	<0.0001	-0.700	<0.0001	0.796	<0.0001	-0.745	<0.0001	-0.778	<0.0001	-0.873	<0.0001	0.149	0.255
miRNA 145	-0.664	<0.0001	-	-	0.954	<0.0001	-0.761	<0.0001	0.710	<0.0001	0.667	<0.0001	0.739	<0.0001	-0.106	0.419
miRNA 155	-0.700	<0.0001	0.954	<0.0001	-	-	-0.741	<0.0001	0.735	<0.0001	0.693	<0.0001	0.756	<0.0001	-0.074	0.572
Intact parathyroid hormone	0.796	<0.0001	-0.761	<0.0001	-0.741	<0.0001	-	-	-0.848	<0.0001	-0.796	<0.0001	-0.849	<0.0001	0.143	0.274
25-hydroxyvitamin D	-0.745	<0.0001	0.710	<0.0001	0.735	<0.0001	-0.848	<0.0001	-	-	0.714	<0.0001	0.714	<0.0001	-0.169	0.197
Flow-mediated dilatation	-0.778	<0.0001	0.667	<0.0001	0.693	<0.0001	-0.796	<0.0001	0.714	<0.0001	-	-	0.839	<0.0001	-0.145	0.270
Estimated glomerular filtration rate	-0.873	<0.0001	0.739	<0.0001	0.756	<0.0001	-0.849	<0.0001	0.714	<0.0001	0.839	<0.0001	-	-	-0.137	0.298
Duration of CKD	0.149	0.255	-0.106	0.419	-0.074	0.572	0.143	0.274	-0.169	0.197	-0.145	0.270	-0.137	0.298	-	-

**Figure 3:**
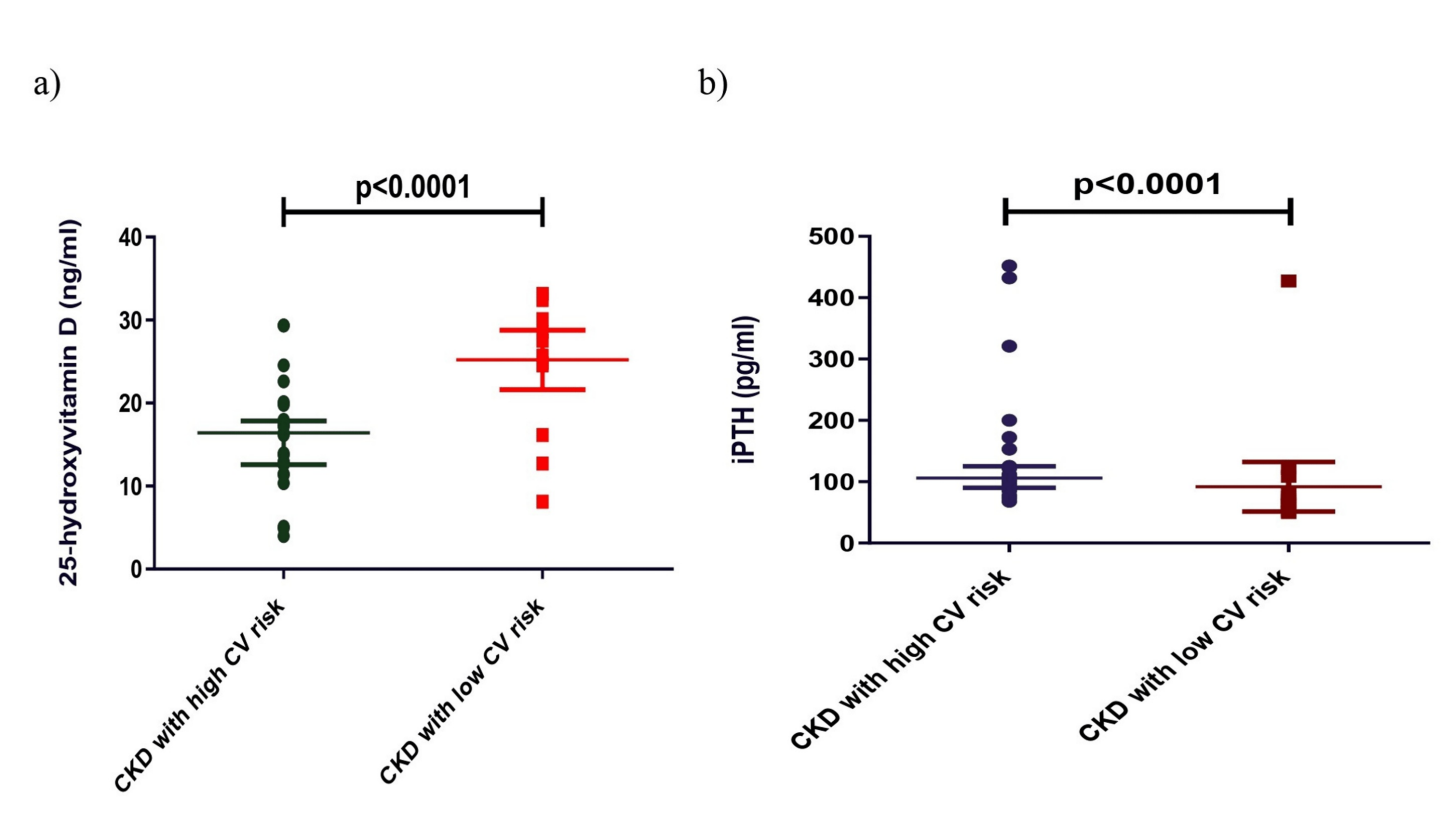
Comparison of 25-hydroxyvitamin D (a) and intact parathyroid hormone in CKD (b) with high CV risk and low CV risk. Mann-Whitney U test was used to analyze these data. CKD: chronic kidney disease, CV: cardiovascular, iPTH: intact parathyroid hormone.

**Figure 4:**
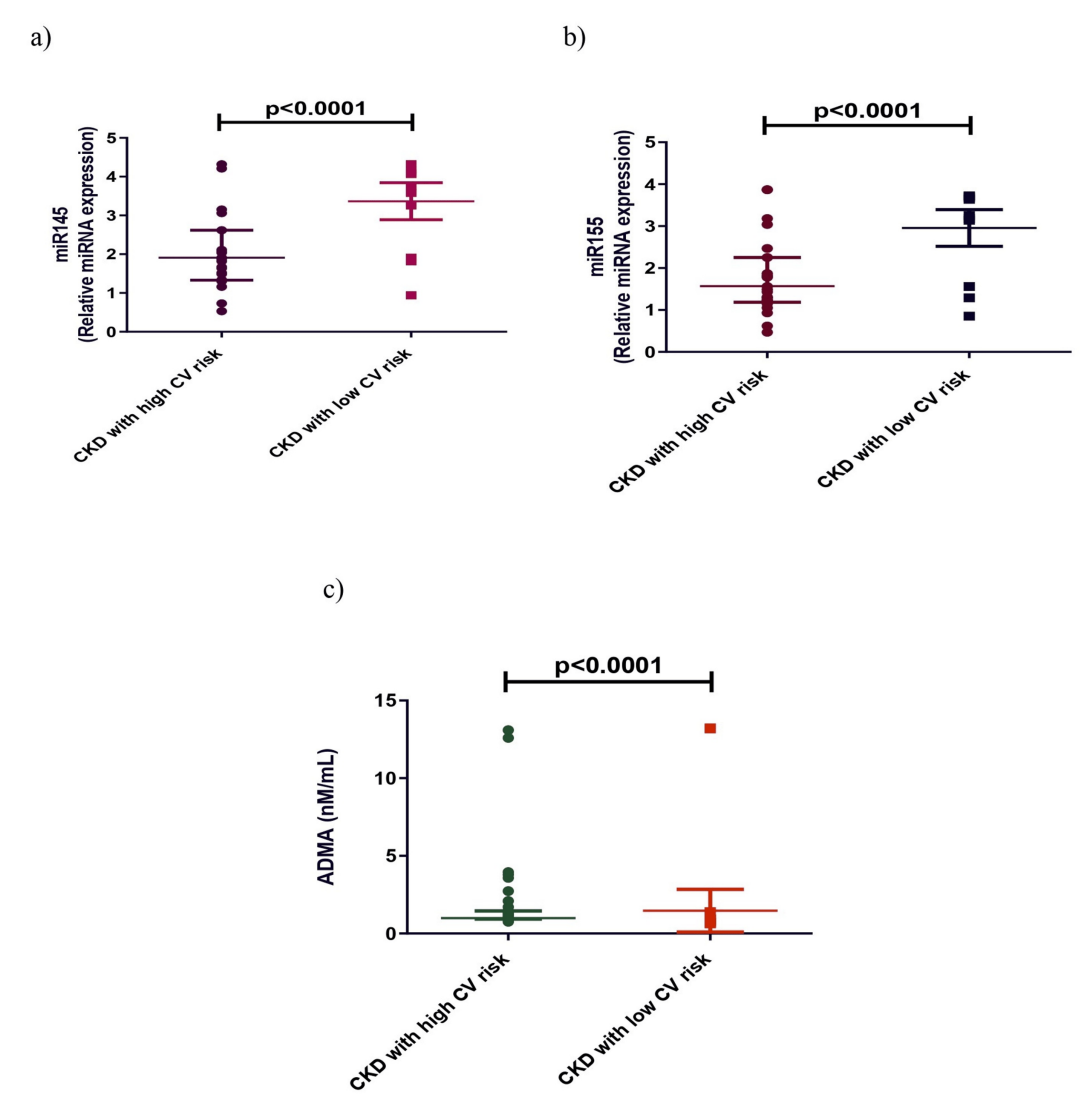
Comparison of miRNA 145 (a), miRNA 155 (b), and ADMA (c) in CKD with high CV risk and low CV risk. Mann-Whitney U test was used to analyze these data. CKD: chronic kidney disease, CV: cardiovascular, ADMA: asymmetric dimethylarginine, miRNA: microRNA.

Additionally, we divided the CKD cases based on high CV and low CV risk groups according to their FMD levels (surrogate marker of CV risk) and found a significant decrease in the 25(OH)D, miRNA 145, and miRNA 155 levels in the high CV risk group. Creatinine, phosphorus, calcium, iPTH, and ADMA levels were significantly increased in the high CV risk group compared to the low CV risk group (Table 4, Figures 3, 4).

**Table 4:** Comparison of study parameters between high and low cardiovascular (CV) risk CKD cases ^ #^Mann-Whitney U test.

Parameter	High CV risk cases (FMD <7) (n=41), median (IQR)	Low CV risk cases (FMD≥7) (n=19), median (IQR)	U-value	p-value^#^
Fasting blood sugar (mg/dL)	79.00 (73.00-86.50)	87.00 (79.00-92.00)	293.50	0.126
Creatinine (mg/dL)	4.00 (3.50-4.00)	2.20 (1.60-2.40)	97.50	<0.0001
Urea (mg/dL)	91.00 (77.50-122.00)	95.00 (83.00-118.00)	343.50	0.465
Total cholesterol (mg/dL)	197.00 (184.00-219.00)	197.00 (177.00-225.00)	384.00	0.930
High-density lipoprotein (mg/dL)	45.00 (38.00-56.00)	55.00 (41.00-61.00)	296.50	0.139
Low-density lipoprotein (mg/dL)	110.40 (100.40-136.50)	108.80 (101.40-129.40)	338.50	0.417
Very low-density lipoprotein (mg/dL)	34.40 (27.20-47.60)	34.60 (27.40-39.60)	369.50	0.750
Triglycerides (mg/dL)	172.00 (136.00-238.00)	173.00 (137.00-198.00)	369.50	0.750
Calcium (mg/dL)	8.90 (8.80-9.25)	8.70 (8.40-9.00)	227.50	0.01
Phosphorus (mg/dL)	6.40 (5.55-6.90)	3.80 (3.60-4.60)	134.00	<0.0001

## Discussion

Cardiovascular problems are the primary risk factors for death in people with end-stage renal failure. In clinical settings, the main effect of medial layer calcification is an increase in arterial stiffness, which can be quite dangerous, especially in large arteries such as the aorta [20]. In our investigation, we discovered that CKD patients had higher levels of phosphate, calcium, and iPTH than controls. We also found a significant increase in creatinine, phosphate, calcium, and iPTH in the high CV risk group compared to the low CV risk group. Our results were concordant with previous studies, where increases in creatinine, phosphate, calcium, and iPTH were associated with elevated cardiovascular risk [21-24].

Extracellular phosphorus binds with calcium and fetuin-A to form calciprotein particles, which are insoluble in water. These highly bioactive particles can induce osteogenic transformation in renal tubular epithelium and vascular endothelium. They are also toxic to cells. Thus, VC and increased blood levels of calcium and phosphate are tightly related [14]. Compared to healthy controls, we observed that CKD patients had lower 25(OH)D levels. A similar result was found in the high CV risk group compared to the low CV risk group. Low 25(OH)D directly inhibits the parathyroid gland and lessens the intestinal absorption of calcium. As a result, PTH levels rise, which is connected to cardiotoxicity-causing left ventricular hypertrophy [25].

Plasma levels of miRNA 155 were considerably lower in CKD patients than in healthy controls. It was also lower in the high CV risk group than in the low CV risk group. The findings of our investigation were consistent with those of Wang et al., who found that individuals with end-stage renal disease and those who had just undergone hemodialysis had considerably lower expression of miRNA 155 [26]. miRNA 155 specifically targets the AT1 receptor, preventing AT1 receptor expression. Thus, in CKD patients, a lower level of miRNA 155 activated the AT1 receptor, which is crucial for renal fibrosis and CVD [17]. Additionally, miRNA 155 regulates the angiotensin-mediated inflammatory response in endothelial cells as well as the transcription of adhesion molecules in these cells [27]. While there was a positive link between miRNA 155 and FMD, we detected a substantial negative correlation between miRNA 155 and ADMA. This is because endothelial cells' eNOS is downregulated by miRNA 155, resulting in less NOS being synthesized, which is crucial for vasorelaxation. Thus, vasodilation is indirectly decreased by miRNA 155. Because eNOS is a potent proapoptotic agent for VSMCs, arterial wall remodeling and increased VSMC proliferation result from its absence [28].

According to our research, CKD patients' levels of miRNA 145 were significantly lower than those of controls. When we compared the markers between low CV risk and high CV risk groups in cases, miRNA 145 levels were lower in the high CV risk group. Taibi et al.'s findings that miRNA 145 levels decrease in atherosclerosis and CKD models, especially in the advanced phases of the disease, corroborated our findings [9]. miRNA 145 controls myocardial transcription to enhance VSMC morphology by blocking an intermediate [9]. Transcription of the primary phenotypic controller of the VSMC myocardium will decrease along with a drop in the miRNA 143/145 complex. This causes VSMCs to change into a more dedifferentiated, proliferating cell, which ultimately results in VC [17].

Our results show a significant difference in indicators of endothelial dysfunction between patients with CKD and healthy controls. Compared to controls, we observed that ADMA levels were considerably higher in CKD patients. We also found that ADMA levels were higher in cases with high CV risk compared to those with low risk. Vo et al.'s results, which showed higher ADMA levels in CKD patients than in controls, corroborated our findings [29]. Atherosclerosis has been connected to the advancement of endothelial cell dysfunction due to ADMA's ability to inhibit eNOS function and decrease NO generation in endothelial cells [4].

When compared to controls, our results demonstrate a substantial decrease in FMD in CKD patients, suggesting that the CKD group has greater endothelial dysfunction. Additionally, Downey et al. demonstrated that FMD was lower in CKD patients than in controls. Severe endothelial dysfunction is present in CKD patients, and this dysfunction worsens as the disease progresses [6]. Endothelial dysfunction occurs in patients with CKD due to diminished NO bioavailability, which can be caused by various factors such as chronic inflammation and the deposition of uremic toxins like ADMA. Increased cardiovascular risk results from reduced NO bioavailability and endothelial dysfunction in CKD [30].

**Figure 5:**
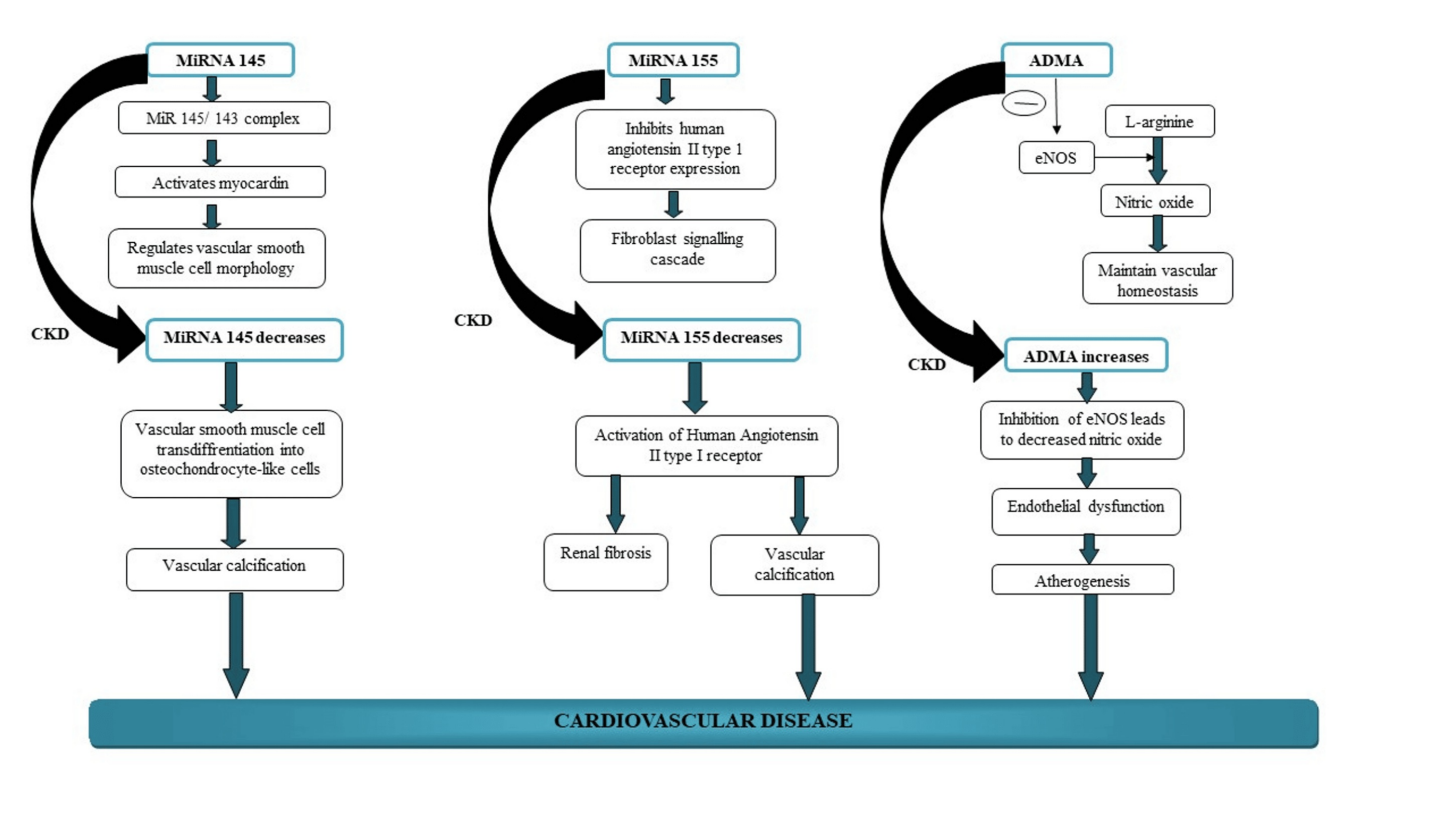
Mechanism of how microRNAs (miRNAs) and asymmetric dimethylarginine (ADMA) are linked to the pathogenesis of cardiovascular disease in patients with chronic kidney disease (CKD)

A noteworthy positive connection was discovered between FMD, eGFR, miRNA 145, miRNA 155, and 25(OH)D. When eGFR is reduced, a major positive correlation is observed between ADMA and iPTH and the severity of the condition. This finding implies that endothelial dysfunction (increased ADMA and decreased FMD) rises with greater CKD severity (reduction in eGFR). As CKD severity increased, there was a corresponding decrease in the levels of miRNA 145 and miRNA 155. This could eventually result in VC and VSMC de-differentiation. The relationship between miRNAs, ADMA, and the pathophysiology of CVD in CKD patients is illustrated in Figure 5. We identified certain limitations in our investigation. Our study was cross-sectional in nature, making it impossible to establish causation or assess the ability of these miRNAs to predict CVD problems in patients with CKD undergoing long-term follow-up.

## Conclusions

Reduced levels of miRNA 145 and miRNA 155, as well as increased endothelial dysfunction, were found to be associated with worsening CKD, suggesting a higher likelihood of progression to CVD. Therefore, future longitudinal studies are needed to assess the potential utility of miRNA 145 and miRNA 155 in predicting future atherosclerosis events in individuals with CKD.
